# Nanomedicine and Phage Capsids

**DOI:** 10.3390/v10060307

**Published:** 2018-06-06

**Authors:** Philip Serwer, Elena T. Wright

**Affiliations:** Department of Biochemistry and Structural Biology, The University of Texas Health Science Center, San Antonio, TX 78229-3900, USA; wrighte@uthscsa.edu

**Keywords:** alpha-sheet, cancerous tumors, capsid dynamics, drug delivery vehicles, native gel electrophoresis, neurodegenerative disease, pathogenic viruses

## Abstract

Studies of phage capsids have at least three potential interfaces with nanomedicine. First, investigation of phage capsid states potentially will provide therapies targeted to similar states of pathogenic viruses. Recently detected, altered radius-states of phage T3 capsids include those probably related to intermediate states of DNA injection and DNA packaging (dynamic states). We discuss and test the idea that some T3 dynamic states include extensive α-sheet in subunits of the capsid’s shell. Second, dynamic states of pathogenic viral capsids are possible targets of innate immune systems. Specifically, α-sheet-rich innate immune proteins would interfere with dynamic viral states via inter-α-sheet co-assembly. A possible cause of neurodegenerative diseases is excessive activity of these innate immune proteins. Third, some phage capsids appear to have characteristics useful for improved drug delivery vehicles (DDVs). These characteristics include stability, uniformity and a gate-like sub-structure. Gating by DDVs is needed for (1) drug-loading only with gate opened; (2) closed gate-DDV migration through circulatory systems (no drug leakage-generated toxicity); and (3) drug release only at targets. A gate-like sub-structure is the connector ring of double-stranded DNA phage capsids. Targeting to tumors of phage capsid-DDVs can possibly be achieved via the enhanced permeability and retention effect.

## 1. Introduction

### 1.1. A Principle

The following principle (basics-focus principle) is proposed here as a foundation for uses of phages in nanomedicine. The curing of currently intractable, biochemically complex diseases requires understanding of the disease basics, but it does not require understanding of the disease details. Historical justifications for the basics-focus principle include (1) the development of smallpox [1] and rabies [2] vaccines before any details about virus composition and structure were known; (2) the discovery of bacterial cell wall-active [3,4] and ribosome-active [5] antibiotics before the composition and structure of bacterial cell walls and ribosomes were known and (3) the use of phage therapy for infectious disease in the absence of knowledge of the composition and structure of phages [6,7]. 

Applying the basics-focus principle does not mean neglecting the rest of the science. Louis Pasteur’s basics-oriented work on improving wine and beer fermentation was a major part of the foundation for the fields of biochemistry and microbiology [2,8]. Antibiotics became major tools in investigating the composition, structure and dynamics of bacterial cell walls [4] and ribosomes [5,9]. 

A proposed corollary is the following. Basics-focus on practical aspects of neurodegenerative and other diseases will not compromise the remaining science. Indeed, as we will describe in Section 6, we think that the remaining science will also be promoted with this approach. 

However, to get started, one has to know enough basics. We think likely that, at least in the case of neurodegenerative diseases, some basics will have to be assumed, without rigorous proof. In the discussion below, we will present both key assumptions and the phage-based evidence in the case of neurodegenerative disease. We will also present a basics-oriented strategy for malignant tumor therapy. This strategy includes use of a phage capsid-based drug delivery vehicle (DDV). 

### 1.2. The Scientific Environment

Articulation of the basics-focus principle is motivated, in part, by a current environment in which progress is politely described as slow for the curing of both neurodegenerative diseases and metastatic cancer. Less politely, but probably more accurately, most (not all) research on neurodegenerative diseases appears to be mired in its focus on complex biochemical details, with only limited symptomatic relief achieved [10,11,12,13]. Focus on these details is the opposite of (1) what has historically been the most successful focus (previous section) and (2) what some fundamentals project to be the optimal focus for developing future therapies for pathogenic virus infections (Section 2.5), neurodegenerative diseases (Section 3.2) and cancerous tumors (Section 4.2). Lack of basics-focus is one possible explanation of why neurodegenerative diseases are incurable at this stage in history.

Two recent books appear to be warning signs that public patience is beginning to exhaust. A recent, cancer-oriented book comes very close to laying the slow-progress blame at the feet of science (really, scientists) [14]. A recent, polio vaccine-oriented book suggests the following. Without the intervention of the law partner of an American President, polio vaccine, as we know it, would not have existed as early as it did [15]. A logical rendition of the current state of phage therapy is likely to exhaust public patience to a new level because, in this case, successful application of the basics has already been achieved ([16], reviewed in [17,18]). However, apparently, in the US, one can receive phage therapy only on an ad hoc basis [16,19]. 

Our entire research history is in the area of phages, with a primary focus on phage assembly. Historically speaking, the Caltech phage group (the home of PS for four years) was, in its early years, supported by the foundation that previously supported the basics focus-oriented work on polio vaccines. This foundation, The March of Dimes (also called the National Foundation), also supported the Pauling-associated work on structure discussed below (see the credits in references [20,21,22,23]). That is to say, the basic philosophy in the current article appears to have an indirect linkage to the distant past.

### 1.3. Phage Assembly Basics 

**Figure 1:**
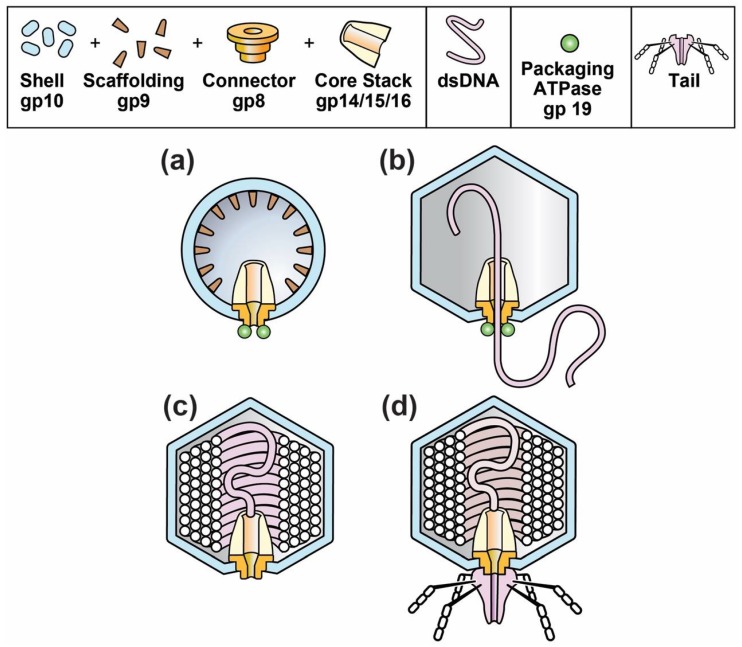
The progression of capsids during the in vivo assembly of phages T3 and T7. (**a**) capsid I; (**b**) capsid II packaging DNA; (**c**) head; (**d**) mature phage.

The composition and structure of phage T3 and its relative, T7, are illustrated in Figure 1d. The phage particle consists of (1) a DNA-encapsulating shell of the protein product of *gene 10*, called gp10; (2) an external structure (tail) adapted for specific binding to host cells and (3) an internal core stack [24,25,26]. The various phage proteins are labeled by gp, followed by the number of the encoding gene. A T3 gene is given the same number as its T7 counterpart [27]. 

**Figure 2:**
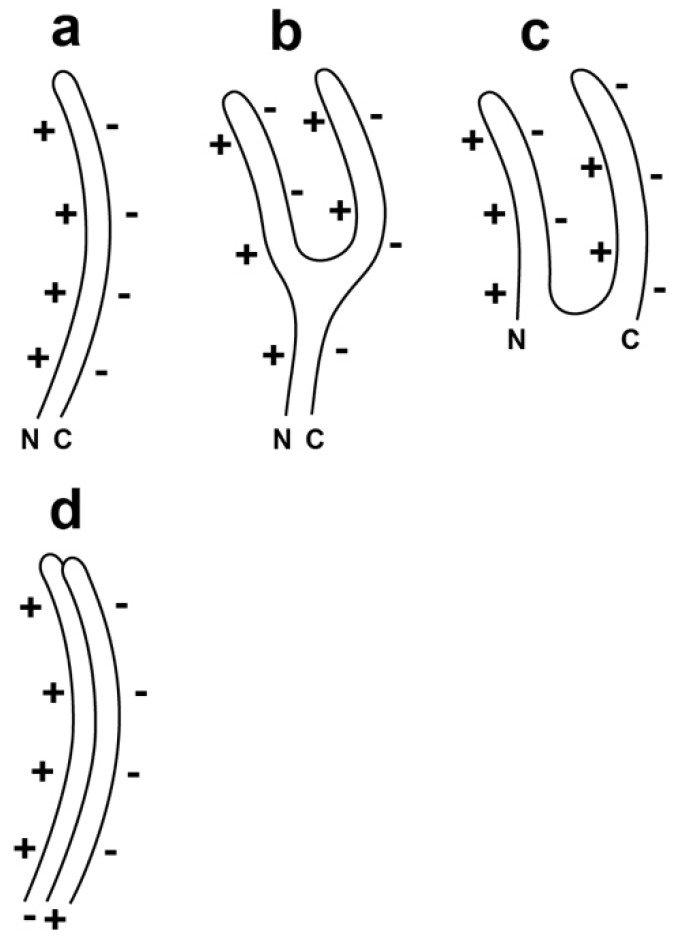
A line drawing of the proposed α-sheet-generating polypeptide backbone of the gp10 subunits of (**a**) hyper-expanded T3/T7 capsid II; (**b**) an intermediate converting from its state in hyper-expanded to its state in contracted capsid II and (**c**) contracted capsid II. N and C indicate the N- and C-terminals of gp10; (**d**) Assembly of two gp10 subunits is shown with the proposed radial staggering. The staggering improves electrical charge-charge-derived energetics. The + symbols indicate the + electrical charge of the α-amino edge; the − symbols indicate the − electrical charge of the α-carboxyl edge.

The DNA-containing, protein capsid of all well-studied, double-stranded DNA phages begins its existence as a DNA-free capsid, called a procapsid. The T3/T7 procapsid (also called capsid I) is illustrated in Figure 1a [24]. The procapsid subsequently packages a DNA genome and, while so doing, changes its structure to form a more phage-like capsid, called capsid II for T3/T7 (Figure 2b). At the end of packaging, the T3/T7 tail is attached to a ring (called portal or connector) that is on a DNA-filled capsid (Figure 2c) called a head [26]. The connector (1) is the site of DNA entry; (2) occupies a 5-fold vertex of an icosahedral, DNA-containing gp10 shell and (3) forms the base of the core stack [24,25]. 

## 2. Phage Assembly and Dynamic States

### 2.1. The Stability of the Icosahedral Shell of the Related Phages, T3 and T7

One’s first impression of the mature T3/T7 capsid is that the DNA-enclosing gp10 shell is extremely stable and unlikely to change states. The shell is resistant to both ionic detergent [28] and the proteases, trypsin and subtilisin [29]. In a mature phage T3/T7 capsid, the major shell protein has a conformation [25] in common among the various double-stranded DNA phages. This conformation is called the HK97 fold, named after the first phage found to have this fold [30].

The mature gp10 shell of T7 capsid II also has a surprisingly high stability to elevated temperature, as discussed in Section 4.3. This characteristic suggests resistance to damage during possible use as a capsid-drug delivery vehicle (DDV). 

Nonetheless, dynamism of phage shells is suggested by cryo-electron microscopy (cryo-EM) analysis. After expulsion of DNA from phage HK97, the shell “bows out” more than it does before expulsion [31]. Similarly, T7 capsid II is found 1.4% larger than the mature capsid when purified [25] and in lysates [32]. If one makes the assumption that HK97-type shell proteins have incompressible components, without capacity for storing and releasing energy, the above result with HK97 is impossible. The reason is that DNA expulsion releases pressure from packaged DNA. This would cause contraction, not expansion, if one makes this assumption. Therefore, the shell of HK97 and probably T3/T7 can move internally to store and release energy.

Less direct evidence for shell dynamics arises from analysis of the leakage of DNA from (tail-free) phage T3 heads. The heads are obtained from a T3 mutant; almost no T3 and T7 heads are without a tail in a wild type infection [26]. The DNA leaks from heads in quantized amounts. This phenomenon is seen via the formation of sharp bands during agarose gel electrophoresis of the DNA remaining packaged after 1-hit restriction endonuclease digestion. The DNA remaining packaged is obtained by (1) DNase I-digestion of external DNA; and (2) expulsion from the capsid of DNA remaining packaged [26]. This leakage-quantization phenomenon is best explained by quantized gp10 shell contraction that evolved via selection for control of the rate of infection-initiating DNA injection [26,33].

In addition, some multi-site T3 mutants undergo enhanced in vivo production of the following gp10-shell variants of capsid II: hyper-expanded and contracted. Identification of hyper-expanded capsid II is made by both (1) electron microscopy and (2) calculation of hydration from the density during buoyant density centrifugation in Nycodenz density gradients of a Nycodenz-impermeable (low density, high hydration) capsid II [34,35]. A DNA-free version of these capsids was initially investigated [34]. 

Next, a DNA-containing version of hyper-expanded capsid II was isolated via its Nycodenz impermeability (sealing) and accompanying low density [35]. This particle underwent contraction in the presence of magnesium ATP, but not magnesium ADP. Binding of ATP to gp10 was proposed to be the source of energy [35]. The following was evidence that the sealing had been evolutionarily selected. For wild type capsid II, cryo-EM revealed the complexity of inter-gp10 subunit interactions to be so high [25] that accidental sealing was improbable. Thus, these capsids were proposed [35] to be in states selected for function during DNA packaging (details [36]). The hyper-expansion required shell thinning to the point that β-sheet was proposed as the likely dominant conformation of gp10 major shell protein [35].

### 2.2. α-Sheet, Rather than β-Sheet, in Size-Altered Capsid II?

α-sheets resemble parallel β-sheets in having a parallel and extended conformation. However, if the amino acids all have the same chirality (as they do in almost all current proteins), then amino acids alternate side chain positions. That is to say, the conformation is not a helical array of amino acids. If constituent amino acids either alternate in chirality or are all glycine, then an α-sheet can be a helical array of amino acids [21,22,23,37,38]. The above has suggested the possibility that α-sheets began existence abiotically, when proteins were glycine-rich and other amino acids involved were not chiral [37,38]. 

An α-sheet-like peptide of 3–6 amino acids is called a nest. Nests are typically glycine-rich. α-sheets and nests have α-amino groups segregated on one edge and α-carboxyl groups segregated on the opposing edge [21,37,38]. Nest-associated α-amino groups are known to bind anions, such as phosphate, via the α-amino group edge. P-loop ATP-binding sites typically have a phosphate-binding nest [37,38]. Thus, one projects that a more extensive α-sheet is also likely to bind phosphates, including those part of ATP.

α-sheets were discovered via model building in 1951 [21] and were originally called parallel pleated sheets before parallel β-pleated sheet was known to be the more frequent structure. However, extensive α-sheets were found, by the discoverers, to be unlikely for the real world of left-handed amino acids. The reason was “steric hindrance between adjacent side chains” [23] for left-handed, non-glycine amino acids. Bending of α-sheets can reduce steric hindrance enough to make extensive α-sheet possible [22]. 

Additional unfavorable energetics are expected from the charge separation of α-sheets in the absence of multiple sheet layers. The α-carboxyl edge is negatively charged; the α-amino edge is positively charged at physiological pH. Indeed, stable proteins have only a small percentage of α-sheet-like nests. Among the proteins stable enough to be characterized, α-sheet-like nests are found primarily in ATP binding sites and in the lining of transmembrane pores [37,38].

The expected increase of α-sheet content for abiotically generated peptides suggests that nests are imprints from times before the existence of living organisms. The idea is that this structure was not completely replaced when increased diversity and chirality arrived for biological amino acids [37,38].

In theory, evolutionary retention of α-sheet structure would be increased if (1) the α-sheet is curved; (2) cooperativity is symmetry-promoted by incorporation of the protein in a symmetrical structure and (3) the protein binds a nest-stabilizing ATP molecule, thereby initiating a cooperative transition to α-sheet structure. Thus, we propose the following hypothesis. During DNA packaging, the observed T3 capsid II shell dynamics (hyper-expansion and contraction) are caused by the adopting by gp10 of ATP-responsive, dynamic α-sheet conformations. 

Specifically, single-layered α-sheet is the proposed structure for gp10 in the shell of the most hyper-expanded version of T3 capsid II (Figure 2a; orientation in the shell is discussed in the next paragraph). The thickness is 0.6–0.9 nm [21,37], which is thin enough to make possible covering of the entire surface of the observed hyper-expanded T3 capsid II [35]. In addition, the proposed structure for the contracted versions of T3 capsid II is multi-layer α-sheet (Figure 2c), generated by an event approximating the folding of the single-layered α-sheet (Figure 2b), without loss of gp10 subunits. This latter conversion would be assisted by favorable electrical charge-charge interactions (Figure 2b). 

Assembly of multiple Figure 2a-like subunits will be inhibited by charge-charge interactions unless the radial positions of neighboring subunits vary so that the negatively and positively charged edges of neighboring subunits are juxtaposed (Figure 2d). This “staggering” of radial position will cause increase in apparent shell thickness when a shell is visualized in a two-dimensional projection of the three-dimensional structure. 

Given the polar nature of the two edges of alpha-sheet, one edge is predicted to be at the outer surface of the capsid’s shell; the other is predicted to be at the inner surface of the shell. Most likely, the negatively charged edge will be at the outer surface to minimize interaction with other intracellular proteins, most of which are negatively charged at neutral pH [39].

### 2.3. Test of a Prediction: Surface Charge 

**Figure 3:**
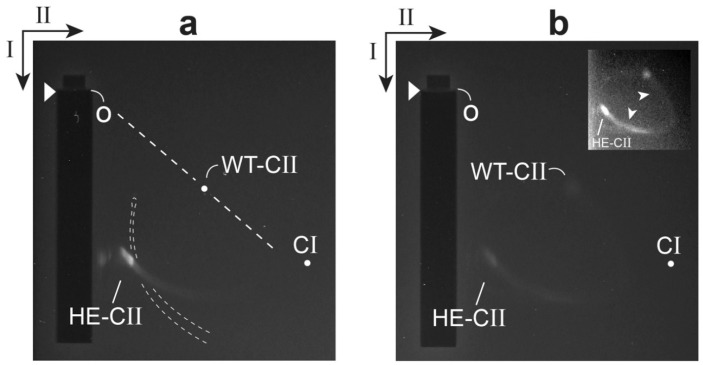
Analysis by 2d-AGE of hyper-expanded T3 ipDNA-capsid II. The ipDNA-capsid II is from the Nycodenz gradient-isolation in Figure 4b of reference [35]. Two fractions of the Nycodenz density gradient were analyzed by 2d-AGE, the (**a**) 1.073 g/mL and (**b**) 1.099 g/mL fractions. The procedure of 2d-AGE is described in reference [26]. The first dimension was run in a 0.30% agarose gel at 2.0 V/cm for 5.0 h. The second dimension was run in a surrounding 2.0% agarose gel at 1.8 V/cm V/cm for 16.0 h. The electrophoresis buffer was 0.09 M Tris-acetate, pH 8.3, 0.001 M MgCl_2_. The temperature was 25 ± 0.3 °C. Seakem LE agarose was used (Lonza, Basel, Switzerland). The arrowheads indicate the leading edges of sample wells. The arrows indicate the directions of the first (I) and second (II) dimension electrophoresis. The curved dashed lines indicate the profile of variable length DNAs (no protein attached) from the DNA fraction of the same Nycodenz gradient. The DNA profile was obtained in a separate quadrant embedded in the same agarose frame as the gels of (**a**,**b**).

We have tested the prediction of a relatively high negative surface charge for the gp10 shell of hyper-expanded T3 capsid II. This was done for capsid II that had incompletely packaged DNA, abbreviated ipDNA; an ipDNA-containing capsid is called an ipDNA-capsid. The test was performed by native agarose gel electrophoresis in two dimensions (2d-AGE) (recent reference [26]): 0.30% agarose gel in the first dimension; 2.0% agarose gel in the second dimension. A band of hyper-expanded ipDNA-capsid II was seen (labeled HE-CII in Figure 3a) after GelStar staining, nucleic acid-specific. This band was also seen after Coomassie staining, protein-specific. The effective origin of electrophoresis is indicated by the letter o. For comparison, the position of wild type T3 capsid II was also determined by 2d-AGE (dot labeled WT-CII in Figure 3a). The latter 2d-AGE was performed in a separate first and second dimension gel embedded in the same agarose frame as the gel in Figure 3a. The position of capsid I was determined from a separate analysis (dot labeled CI). 

The effective radius of a particle (*R*_E_), together with the radius of the gel’s effective pore (*P*_E_), uniquely determine the straight line drawn from the effective origin to the position of a particle in the gel. This line (dashed) is indicated for the WT-CII position in Figure 3a (*R*_E_ = 28.6 nm by small angle X-ray scattering [40]). The angle, θ, decreases as *R*_E_ increases [26]. Thus, the particles of the HE-CII band are confirmed in Figure 3a to be relatively large. The shape and orientation of HE-CII band indicate that these particles are also heterogeneous in *R*_E_. 

The average particle of the HE-CII band also had an average electrical surface charge density, σ, that was negative and was increased in absolute value. The reasoning is the following. The value of σ has, in general, been found to be proportional to the electrophoretic mobility in the absence of a gel. This mobility has been found to be independent of internal contents, such as ipDNA ([26] and included references). The ratio of σ value for HE-CII to σ value for WT-CII was 1.9. This ratio was determined from the ratio of average distances migrated in the first (low sieving) dimension, corrected for an estimated 5% greater effect of sieving on HE-CII in the first dimension. When CI was substituted for HE-CII, this ratio was also 1.9. 

The source of the relatively high negative σ of hyper-expanded ipDNA-capsid II had to be either (1) the σ at the surface of shell-associated gp10 or (2) leakage from the capsid of a segment of (negatively charged) ipDNA, without dissociation of the ipDNA. We concluded that the former possibility was correct because electron micrographs did not reveal any leaked DNA [35]. Thus, qualitatively, the above prediction was confirmed. 

Finally, the relatedness of HE-CII and WT-CII particles was confirmed in the 2d-AGE profile of a higher density fraction from the same Nycodenz density gradient. In this case, the HE-CII band was connected to a WT-CII-like band by an arc formed by capsid II particles with intermediate *R*_E_ and σ values (Figure 3b and inset). Contrast enhancement was used in the inset of Figure 3b to make the arc more easily seen. Presumably, the arc-forming particles were also intermediate in structure. 

### 2.4. Electron Microscopy

**Figure 4:**
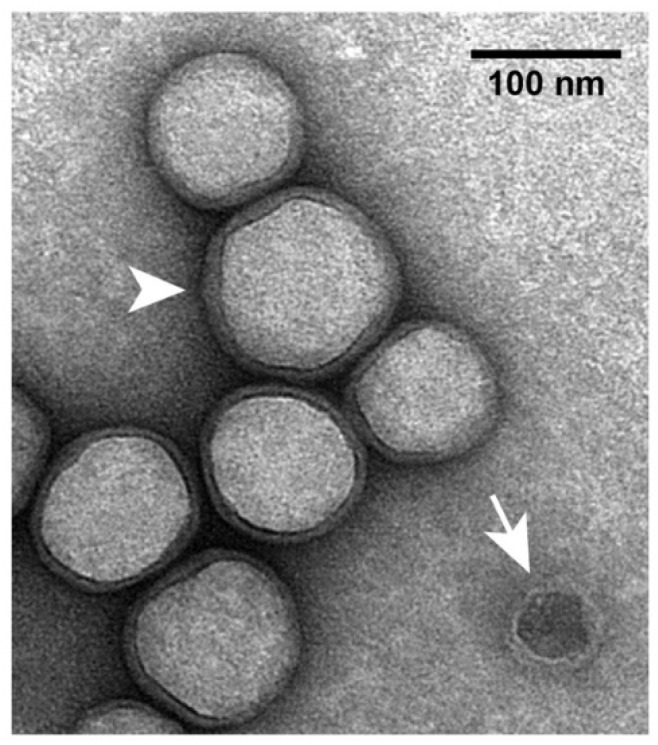
Electron microscopy of hyper-expanded NLD capsid II. The sample was the same as the sample used for Figure 2a. Particles in the sample were negatively stained with 1% uranyl acetate. The procedures of specimen preparation and EM were the same as used in reference [35].

In previous electron micrographs of specimens negatively stained with sodium phosphotungstate, hyper-expanded ipDNA-capsids appeared full [35]. This appearance was caused by impermeability to the negative stain, not by filling with DNA [35]. The low-electron density interior was occupied by dried buffer components. The appearance was similar after negative staining with uranyl acetate (Figure 4). However, the following feature was exaggerated in some particles of hyper-expanded NLD capsid II. A thin, dark layer of negative stain separated the light interior from the light gp10 shell (Figure 4). Apparently, after drying of most of the interior, negative stain leaked through the gp10 shell and then dried in a thin layer. In contrast, a contaminating wild type capsid II-like particle had the traditional negative stain-filled appearance (arrow in Figure 4). The shell of the latter capsids is 2 nm thick. 

In the shell-revealing regions of hyper-expanded ipDNA-capsid II particles, the shell usually appears 3–5 nm thick. However, the thickness of the shell is likely to be significantly less than that (Section 2.2). This difference is enough so that a staining alone is unlikely to be the cause. Thus, the cause of the above difference resides in changes to the gp10 shell that occur in the latter stages of negative staining. 

Details were suggested by the observation that, in some regions, the gp10 shell appeared doubled (arrowhead in Figure 4). The radial position staggering proposed in Figure 2d explained the observed doubling via an increase in the staggering distance at the latter stages of negative staining. By this explanation, embedding in the negative stain limited shell disruption to sub-observable levels. The regions of apparent shell thickening, without doubling, could be explained by smaller increase in the staggering distance and superposition of images from multiple planes perpendicular to the direction of observation. In summary, the electron microscopy produced images that were explained by a gp10 structure similar to the one in Figure 2d. Higher resolution, direct determination of structure is needed to test more directly for α-sheet and other structures. 

### 2.5. Possible Pathway to Anti-Viral Therapeutics

The above analysis by 2d-AGE has been performed only for phage T3. This analysis is also a relatively simple, inexpensive, sensitive way to determine whether other viruses produce size-altered capsids like those of phage T3. For now, we make the following extrapolation. Pathogenic viruses do make extensive α-sheet-containing shell intermediates, at least in the case of the capsids of viruses with a double-stranded DNA genome, such as herpes viruses. 

If, indeed, this is true, then the following is a possible strategy for anti-viral therapy. Find or design therapeutic compounds that target the backbone of extensive α-sheets. The low frequency of extensive α-sheets suggests low toxicity. This strategy has the projected advantage of being insensitive to bypass via single mutations. In addition, a relatively broad spectrum of viruses is likely to be susceptible. Two major factors in reducing the success of anti-viral drug therapies are evolution of resistant viral mutants and presence of narrow drug specificity (examples [41]). 

## 3. Dynamic States and Neurodegenerative Disease

### 3.1. Some Details

Neurodegenerative diseases are all characterized by the presence of protein aggregates collectively called amyloid. This name is derived from starch-like texture. The aggregate-forming protein varies with the neurodegenerative disease (reviews: ALS [42], Alzheimer [43], Huntington [44], Parkinson [45], prion-associated [46]). When various proteins form amyloid, they convert from an original mixed-element structure to a predominantly β-sheet structure. 

Although β-sheet is the predominant conformation of accumulating amyloid protein, computer simulations reveal that α-sheet is (1) accessible to amyloid-forming proteins [47,48] and (2) selectively accessible to them at low pH, especially in the protein region thought to initiate the transition to amyloid [48]. The proposal has been made that charge-charge interactions assist assembly to form an α-sheet intermediate that subsequently converts to β-sheet during the formation of amyloid. Molecular dynamics simulation has shown the possibility of conversion of α-sheet to β-sheet [47,48,49]. 

Given the association of nest-like structures with membrane pores, the theory is that extensive α-sheet-containing amyloid proteins, which are minority species, generate toxicity by making pores in cell membranes [37,38,47,48]. Mature amyloids are complex enough so that the amount of α-sheet associated with the β-sheet structure is, to our knowledge, not known.

Returning to the biology, the hypothesis has previously been proposed [50] (and expanded [51]) that one of the normal (non-toxic) functions of amyloid-forming proteins is innate immunity. This innate immunity occurs via neutralizing of products of both pathogen-generated and physical insults. By this hypothesis, neurodegenerative disease occurs when control of this insult-neutralizing activity is lost and, in some cases, the innate immune proteins start neutralizing themselves. A result is accumulation of toxic aggregates. Indeed, more recent studies have shown anti-bacterial and anti-viral effects of the amyloid for Alzheimer disease [52,53].

The expanded proposal [51] originally was that the neutralizing interaction occurred via the extension by innate immune proteins of β-sheet structure generated by the insult. However, the above considerations suggest substitution of α-sheet structure for β-sheet structure. 

### 3.2. A Proposal for Reduction to Practice

We propose the following working assumptions to implement the basics-focus principle for neurodegenerative diseases. (1) Pathogenic viral infection and other insults trigger activation of amyloid-forming host innate immune proteins. These proteins convert to extensive α-sheet structure and, then, co-assemble with and inactivate extensive α-sheet-rich structures generated by the insults. These activities are currently obscure; (2) Extensive α-sheets are rare to non-existent in endogenous proteins of the virus host, other than activated innate immune system proteins; (3) Neurodegenerative diseases are caused by α-sheet-dependent damage caused by hyper-produced, activated innate immune proteins. These proteins eventually form β-sheet-rich amyloid. 

Other virus-induced innate immunity activities (cytokine-induced neuroinflammation, for example) are known to be associated with the onset of neurodegenerative disease [54]. By the above hypothesis, these activities are secondary.

The proposal of (1)–(3) includes the fundamental idea that higher organisms have retained an immune system that has anciently derived targets and mode of action. This system is based on an abiotically derived protein structure. This system is difficult to analyze because proteins with more recently evolved composition and structure force both the innate immune system and its targets into intermittent status. 

A way to reduce these ideas to practice is the following, as similarly suggested [51] before the introduction of the above α-sheet proposal. (1) Select for low-pathogenicity viruses that yield unstable, α-sheet-rich complexes when innate immune system proteins co-assemble with intracellular intermediates of this virus; (2) Infect patients with these viruses. To manage adaptive immunity, several such viruses would be used in succession. To isolate the needed viruses, one could begin by trying to find cases of spontaneous (presumably limited) disease-remission that have a correlation with a previous virus infection. The correlation would presumably not be dramatic and finding it would require determination. The reason is that most infections are expected to have the opposite effect, i.e., to trigger disease [54,55]. Therefore, the desired post-infection remissions are likely to be rare. 

Of course, selecting for any immune system-avoiding virus has an obvious potential danger of ending with a problem of virus virulence. Presumably, virus virulence would be carefully monitored during selection. In any case, the selection would include screening for low virulence. Low virulence might, indeed, be necessarily co-selected during selection for an immune complex-disrupting phenotype. 

## 4. Phage Assembly and Development of Gated, Targeted Drug Delivery Vehicles

### 4.1. Avoiding Immune Systems

Here, we change direction to discuss use of phage capsids as DDVs for the therapy of cancerous tumors. Administration of phage capsids is accompanied by the problem of phage capsid removal/neutralization by host immune systems. Both adaptive and innate immune systems will act to remove or neutralize foreign nanoparticles, including phages [56]. Optimization of a DDV-based strategy must include a response to this problem. One response to adaptive immunity is to serially change the phage-source of the DDV, in analogy to what is proposed above for low-virulence, therapeutic, eukaryotic viruses. In addition, phages propagate rapidly enough so that directed evolution can be used to reduce removal of a phage-derived DDV by any immune system. 

However, probably the most significant potential advantage of a phage capsid-DDV is gating via the connector (formed by gp8 for T3/T7; Figure 1). The connector of phages T3 and T7 also exists for all other studied double-stranded DNA phages (reviews [57,58]). Thus, if connectors can, in general, be used as gates to capsid-DDVs, nature will provide many immunologically unrelated DDVs. 

Before adaptive immunity becomes limiting (which takes 1–2 weeks in the case of phage K [56]), innate immune systems reduce effectiveness of any unprotected nanoparticle. Apparently the most active system is the mononuclear phagocyte system (MPS), formerly called the reticuloendothelial system. The MPS moves nanoparticles primarily to the liver and spleen [59,60]. Published tests of the efficiency of the MPS have been made with phages lambda, P22 and K. Phages lambda and P22 are lysogenic. When in a lysogenic state, lambda and P22 presumably could not have experienced and adapted to their environments. In contrast, phage K is lytic and had continuously experienced and adapted to its environment. Therefore, one expects that past genetic adaptation would cause phage K to have a longer lifetime in mouse circulation. This is the case. Complete removal occurs in over 24 hr for phage K [56] and 3–4 h for phages lambda and P22 [61,62]. 

That the above difference in lifetimes can be adaptive for phage lambda was shown by 10-cycles of the following: mutagenic growth, followed by mouse passage [61]. The adapted lambda was removed from mouse circulation more slowly than wild type lambda. Removal was by approximately a factor of 10 per day, several orders of magnitude more slowly than before adaptation [61]. A comparable removal rate was found for lytic phage T3 without any laboratory adaptation [63]. Apparently, the needed adaptation had occurred for T3 in the wild. Systematic attempts to make lytic phages more long-lived in circulation have not been made to our knowledge. 

### 4.2. Targeting Tumors

Perhaps the most dramatic potential application of the basics-focus principle is to the therapy of cancerous tumors, including metastatic tumors. Focus on details (rather than basics) leads to targeting of either tumor-concentrated biochemistry or a patient’s immune systems. However, the biochemistry of tumors overlaps the biochemistry of normal cells. Thus, essentially all drug and radiation therapies are toxic, which limits the extent of the therapy and makes patients sick [64,65,66]. Multi-faceted toxicity of immunotherapy is emerging as a problem [67]. In addition, when a cancer becomes metastatic, none of the above therapies can systematically block bypass of the therapy via evolution of cancer cells. 

However, tumors do have one targetable, basic characteristic that they apparently cannot evolve to eliminate. This characteristic is the presence of 10–200 nm-sized pores in the blood vessels that feed tumors. Healthy blood vessels do not have these pores [68,69].

Thus, one should be able to achieve specific drug delivery to tumors with the following strategy: use of a DDV small enough to fit in these pores, but too large to pass through healthy blood vessels. In addition, tumors have relatively poor lymphatic drainage. Thus, when a DDV enters a tumor, it is slow to leave. The combined effect of the pores and the poor drainage is called the enhanced permeability and retention (EPR) effect [68,69]. 

Knowledge of the EPR effect is not new. The EPR effect has been the foundation for improved drug delivery via liposomal DDVs. Several products are FDA approved [70], the earliest being Doxil [71]. However, results have been disappointing. A recent review indicates the following limitations [72]: (1) “rapid loss of the drug cargo … often immediately after … systemic administration”; (2) “in many cases, less than 1% of the administered nanoparticle dose reaches the malignant tissue”; (3) “lack of release of drug in tumors”; (4) safety of the DDV; (5) safety of impurities (bacterial endotoxin which can generate a cytokine storm); (6) immunotoxicity (complement-generated, for example) and (7) standardization during manufacturing scale-up. A major uncertainty is the extent of the EPR effect in early metastases. 

In evaluating the use of a phage capsid-DDV, we initially note that phages have never been found to be toxic to humans. Phages T3 and T7 were presumably isolated from sewage [73] and have passed through humans many times with the associated likelihood that they and their capsids already have evolved to avoid human immune systems. Furthermore, phage capsids with DDV potential are assembled in vivo and easily purified with structure uniform enough to obtain a 3–4 Å cryo-EM structure of the shell in the case of T7 [25]. If gated, a phage capsid is well on its way to removing the above limitations.

Indeed, one form of T3 capsid II is sufficiently gated so that it does not allow Nycodenz (molecular weight = 821) in its internal cavity until the temperature is raised. That is to say, the elevated temperature opens a gate. The gate is closed by lowering temperature, with the loaded Nycodenz not leaking detectably. The connector is the likely location of the gate [74].

### 4.3. Adequate Loading of a Phage Capsid-DDV

Nonetheless, a major barrier still exists to implementation of a gated T3 phage capsid-DDV. When we used elevated temperature to increase permeability (open the gate) in the presence of 10 mg/mL doxorubicin, the T3 gp10 shell was damaged. Native gel electrophoresis suggested disassembly to small aggregates, possibly monomers of gp10 (unpublished data). This limitation was possibly caused by detergent characteristics of doxorubicin, which has a positively charged and a hydrophobic region. These two regions are also present in most anti-cancer drugs. 

Although this limitation has not yet been bypassed, we have found that the T7 counterpart of the loadable T3 capsid II is much more stable (80–83 °C). Future work will focus on the T7 version of this capsid II.

## 5. Prospects for the Future

Hypothesis-derived theory and practice can differ, possibly because of variables not in the theory. Also, hypotheses can be incorrect. The above proposals for treating neurodegenerative disease and cancerous tumors are not exempt from this pattern. Nonetheless, when basic considerations point in a simple direction, attempts to go in that direction should, it seems to us, be made. However, in the absence of a change in priorities, the probability is very close to zero that the above strategies will be tested experimentally within the next few years. 

As support for this pessimism, exhibit A is the current status of phage therapy of infectious disease. For phage therapy, the basics point in a clear direction (details [17]). In addition, going in this direction is supported by (1) historical [6,7,73] and present-day [16] examples of dramatic success of doing that and (2) recent development of technologies that should dramatically improve results, if deployed. These include technologies of computerized database use/phage storage/phage retrieval, in addition to updated procedures of phage isolation and characterization. Yet, few, if any, systematic attempts at implementation are being made. Phage therapy is performed on an ad hoc basis [16,75], which slows implementation, sometimes to the point that it is too late.

## 6. Relationship to Scientific Details

In Section 1.1, we gave examples of scientific output triggered by de facto past clinically oriented use of the basics-focus principle. The finding of a curative, low-virulence virus, as proposed above, should do the same in the case of neurodegenerative diseases. Existence of such a virus would likely trigger experiments that use the virus to determine the interaction of virus metabolism and assembly with cellular events. Cellular events would be better understood. One finding might be that the above assumptions need to be modified or abandoned. However, the overall direction embodied in the assumptions could still be accurate enough to obtain a cure. 

A gated, uniform-size, uniform-structure DDV can also be used to track the progression of tumors via the loading of the DDV with a trackable compound. Nycodenz, for example, is x-ray opaque and can be tracked by use of micro-CT. Such tracking would be used both clinically and also to analyze pathways of receptor mediated endocytosis and lysosome targeting of endocytic vesicles. 

In other words, the division between basic science and clinical practice is not sharp.
